# Orientin Reduces the Effects of Repeated Procedural Neonatal Pain in Adulthood: Network Pharmacology Analysis, Molecular Docking Analysis, and Experimental Validation

**DOI:** 10.1155/2023/8893932

**Published:** 2023-11-24

**Authors:** Dong-Dong Guo, Hai-Yan Huang, Hai-E. Liu, Kun Liu, Xing-Jing Luo

**Affiliations:** ^1^Department of Anesthesiology, National Children's Medical Center, Children's Hospital of Fudan University, Shanghai 201102, China; ^2^Department of Cardiovascular, Shanghai Municipal Hospital of Traditional Chinese Medicine, Shanghai University of Traditional Chinese Medicine, Shanghai 200071, China

## Abstract

**Background:**

Premature infants often undergo painful procedures and consequently experience repeated procedural neonatal pain. This can elicit hyperalgesia and cognitive impairment in adulthood. Treatments for neonatal pain are limited. Orientin is a flavonoid C-glycoside that has repeatedly been shown to have pharmacological effects in the past decades. The aim of this study was to systematically explore the effect of orientin on repeated procedural neonatal pain using network pharmacology, molecular docking analysis, and experimental validation.

**Methods:**

Several compound-protein databases and disease-protein databases were employed to identify proteins that were both predicted targets of orientin and involved in neonatal pain. A protein-protein interaction (PPI) network was constructed, and Gene Ontology (GO) and Kyoto Encyclopedia of Genes and Genomes (KEGG) enrichment analyses were performed to explore the potential mechanism of action. Molecular docking analysis was employed to calculate the binding energy and visualize the interactions between orientin and potential target proteins. Finally, a mouse model of repeated procedural neonatal pain was established and orientin was administered for 6 days. The mechanical and thermal pain thresholds were assessed in neonates and adult mice. A Morris water maze was employed to investigate cognitive impairment in adult mice.

**Results:**

A total of 286 proteins that were both predicted targets of orientin and involved in neonatal pain were identified. The hub proteins were SRC, HSP90AA1, MAPK1, RHOA, EGFR, AKT1, PTPN11, ESR1, RXRA, and HRAS. GO analysis indicated that the primary biological process (BP), molecular function (MF), and cellular component (CC) were protein phosphorylation, protein kinase activity, and vesicle lumen, respectively. KEGG analysis revealed that the mitogen-activated protein kinase (MAPK) signaling pathway may be the key to the mechanism of action. Molecular docking analysis showed the high binding affinities of orientin for MAPK1, MAPK8, and MAPK14. In mice, orientin inhibited the hyperalgesia in the pain threshold tests in neonates and adult mice and cognitive impairment in adult mice. Immunofluorescence showed that phosphorylated MAPK1 (p-ERK) protein levels in the hippocampus and spinal dorsal horn were downregulated by orientin.

**Conclusion:**

The findings suggested that orientin alleviates neonatal pain, and the MAPK signaling pathway is involved.

## 1. Introduction

The vital signs of premature infants (born before 37 weeks of pregnancy) are often poor worrying, so they require a neonatal intensive care unit stay (mean duration: 25 days) and 10–18 painful procedures every day according to reports in the literature [1–3]. Neonates can feel pain, and the pain may elicit several immediate and long-term neurobehavioral abnormalities, including changes in pain perception and cognitive impairments in adulthood [4–6]. Treatments in clinical practice for repeated procedural neonatal pain are opioid and nonopioid analgesics (acetaminophen and nonsteroidal inflammatory drugs), but all abovementioned drugs must be carefully administered due to underdeveloped renal function in newborn [7] and few reported further changes in adulthood. Are there any drugs that can improve immediate and long-term neurobehavioral abnormalities induced by neonatal pain? Ranger et al. reported a failed attempt at using sucrose in a mouse model of repeated procedural neonatal pain [8]. Paracetamol only inhibited long-term behavioral effects, but not repeated procedural neonatal pain [9].

Orientin is a water-soluble flavonoid C-glycoside [10] that belongs to the family of flavonoid glycosides [11]. Orientin widely existed in plants worldwide, for example, *bamboo* [12], *Celtis africana* [13], and *Jatropha gossypifolia* [14]. In the past decades, it has been shown to have multiple pharmacological effects, such as anticancer [15], antiviral and antibacterial [16], antioxidant [17], and anti-inflammatory [18] effects. Due to its anti-inflammatory and antioxidant effects, it exerts many protective effects, including protection against myocardial infarction [19], radioprotection [20], neuroprotection [21], attenuation of cerebral ischemia/reperfusion injury [22], and inhibition of high-glucose-induced apoptosis involving mitophagy [23]. We previously revealed the analgesic effect of orientin in neuropathic pain [24]. Although it is a widely-recognized low-toxic [10], water-soluble flavonoid, whether it can treat neonatal pain is unknown.

Unlike previous predictions of the mechanisms of action of drugs, which relied on experiments, network pharmacology analysis to predict mechanisms does not require experimentation, and this field has been developing rapidly, especially in the natural product field [25]. Network pharmacology is based on systems biology and bioinformatics and can involve high-throughput screening and the construction of multimolecular, multitarget, and multilink “drug-compound-target” network models, which reveal multilevel information [26]. Furthermore, molecular docking analysis (based on artificial intelligence software) can be used to indicate possible interactions between a compound and its target proteins, the possible amino acid sequence length, and types of binding amino acids [27]. This study aimed to systematically explore the effect of orientin on repeated procedural neonatal pain using network pharmacology analysis, molecular docking analysis, and experimental validation. Target proteins of orientin that are involved in neonatal pain were used in a protein-protein interaction (PPI) network and the key proteins were then identified. A key signaling pathway involved in the mechanism of action was predicted, and the predicted mechanism was validated *in vivo*.

## 2. Materials and Methods

### 2.1. Prediction of Target Proteins

The network pharmacology analysis was performed following previous reports [28, 29]. The target proteins of orientin were predicted by using The Chinese Traditional Medicine System Pharmacological Database and Analysis Platform (TCMSP) [30], SwissTargetPrediction database [31], and SuperPred database [32] by using the term “orientin.” The target proteins involved in neonatal pain were obtained using the GeneCards database [33], DisGeNEt database [34], and DrugBank database [35] using the term “neonatal pain.” Duplicates were removed and the canonical protein names were determined using the UniProt database [36] and then inputted into Venny 2.1.0 [37] to identify the overlapping proteins and to create a Venn diagram.

### 2.2. Protein-Protein Interaction (PPI) Network

The overlapping proteins were submitted to the STRING database [38] to create a PPI network and to detect the links and control hubs. The species was limited to “*Homo sapiens*” and the minimum required interaction score was set at >0.7 (high confidence) [39]. The PPIs were downloaded into Cytoscape software v3.8.2 [40], which is commonly used for visualization in complex networks. The cytoHubba plugin [41] was used to determine the top 10 hub proteins based on degree.

### 2.3. Gene Ontology (GO) and Kyoto Encyclopedia of Genes and Genomes (KEGG) Enrichment Analyses

Metascape [42], an automatic bioinformatics tool, was used to subject the overlapping proteins to a GO analysis of enriched gene functions, involving molecular functions (MFs), biological processes (BPs), and cellular components (CCs). A KEGG analysis was also performed; the results were downloaded and the top 20 pathways were automatically visualized using Metascape.

### 2.4. Molecular Docking Analysis

To further validate the network pharmacology results, which predicted protein targets of orientin to treat neonatal pain, a molecular docking analysis of orientin and key target proteins was performed. In brief, a 2D structure of orientin was downloaded from the PubChem database and converted to a 3D structure using ChemBio3D mol2 software after minimizing the energy. The 3D structures of the target proteins were downloaded from the Protein Data Bank (PDB) and any ligands were removed. AutoDock v1.5.7 was employed for ligand preparation, water removal, nonpolar hydrogen atom preparation, and locating the active pocket. AutoDock Vina [43] was used to determine the final docking conformation. The binding amino acids in the proteins and the conformation with the best binding affinity were visualized using PyMOL Molecular Graphics System v2.0 (Schrödinger, LLC, Germany) [44] in the Python environment.

### 2.5. Animal Grouping and Model Establishment

Ethics approval (grant no. 2022007) was obtained from the Laboratory Animal Ethics Committee of the Shanghai Municipal Hospital of Traditional Chinese Medicine of the Shanghai University of Traditional Chinese Medicine. Institute of Cancer Research (ICR) mice were provided by Shanghai Jie-si-jie laboratory Animal Co. Ltd (Shanghai Lab Animal grant no. SCXK (H) 2018-0004).

**Figure 1:**
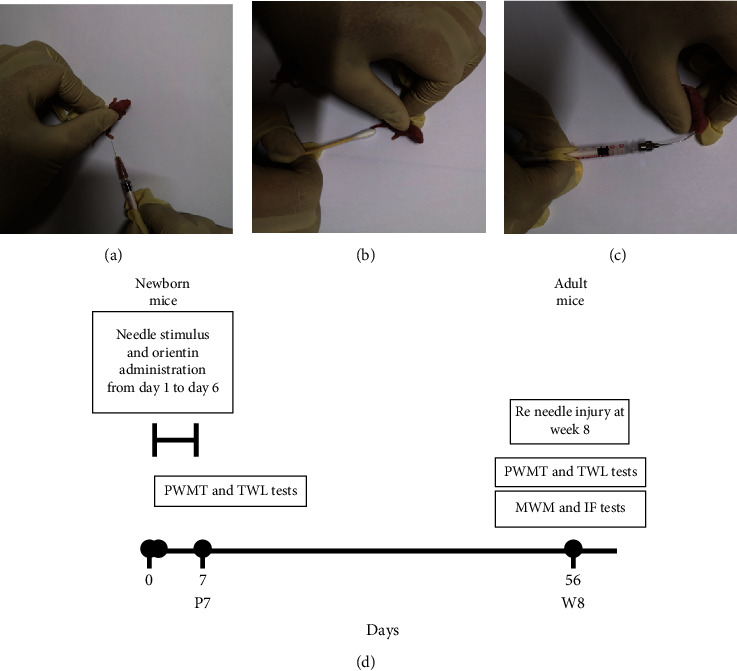
The model establishment. (a) Operation in the model group. (b) Operation in the sham group. (c) Administration in orientin groups. (d) The flow chart of animal studies.

A review concerning children born prematurely who are subjected to neonatal repeated procedural pain is linked to changes in cognitive, pain threshold and psychosocial function such as vulnerability to stress disorders in adulthood life [45]. To mimic NICU pain in a preterm neonate due to repeated procedures, a repetitive needle-pricking rat model was employed in 2012 [46]. Pups in the model group received needle prick several times while pups in the tactile control group received gentle tactile stimulations. After that, this model was widely used for the evaluation of the effectiveness of drugs and interventions [8, 47]. In this study, a model of repeated procedural neonatal pain was established by mainly following the method reported by Ranger et al. [8]. After obtaining pregnant mice and after delivery of the pups, 1-day-old pups (P1) were randomly assigned to six groups (*n* = 6 per group): (1) control group (no stimulation or treatment), (2) model group (a needle was used to prick a hind paw, and sterile water was administered orally (Figure 1(a))), (3) sham group (a cotton-tipped swab was applied to a hind paw to create tactile pressure (Figure 1(b))), and (4–6) three orientin groups (stimulus same with the model group, and oral orientin at 7, 14, or 21 mg/kg was administered (Figure 1(c))). The stimuli and orientin were administered from P1 to P6. The stimuli were administered 10 times per day, while orientin was administered 4 times per day (AM 8, AM 12, PM 4, and PM 8). After P6, there were no orientin administrations.

Some studies added a reinjury at 8 weeks to observe changes in pain threshold during adulthood because neonatal repeated procedural pain leads not only to acute short-term hyperalgesia but also to changes in pain threshold in adulthood [9, 48]. In this study, a reinjury was also established following previously described methods [9, 48]. In brief, at week 8 (W8), for each relevant mouse, a hind paw was pricked with a needle. No drug was administered this time. The flowchart is shown in Figure 1(d).

### 2.6. Paw Mechanical Withdrawal Threshold (PWMT) and Thermal Withdrawal Latency (TWL)

PWMT was assessed using calibrated von Frey filaments (Stoelting, Kiel, WI, USA) at P7 and W8. The mice were placed on a metal mesh floor in a chamber. After accommodation, a von Frey filament (0.04, 0.07, 0.16, 0.4, 0.6, 1.0, and 1.4 g) was pressed perpendicular to the plantar surface of the hind paw until it bent [49]. The minimum force required to induce three positive withdrawal responses (withdrawal or contraction) in five attempts using the von Frey filament (with an interval >1 min) was recorded [50]. If the maximum stimulation intensity (1.4 g) did not produce a withdrawal response, the force was recorded as 1.4 g. Tests were conducted three times (with a minimum interval of 1 h), and the mean force was recorded as PWMT.

TWL was assessed at P7 and W8. The mice were placed on a hot plate at 52.5°C (IITC Life Science, Woodland Hills, CA, USA) [50]. The response latency to elicit a positive withdrawal response (e.g., withdrawal, licking, retraction, or jumping) was recorded. If a positive response was not elicited, the mice stayed on the hot plate for 30 s at most (to avoid scalding and injury) and the TWL was recorded as 30 s. Tests were conducted three times (with a minimum interval of 1 h), and the mean value was recorded as TWL.

### 2.7. Morris Water Maze (MWM) Test

The MWM test was employed to investigate the effect of orientin on hippocampal-dependent spatial reference memory in adult mice at W8 [51]. The test was performed using a video analysis system (XR-XM-101, XinRuan Corporation, Shanghai, China), a round gray water pool (height: 1.2 m, weight: 0.5 m; divided into four quadrants according to the four directions of northeast, southeast, southwest, and northwest), a small underwater platform (height: 29 cm), and a tracking camera positioned directly over the pool. To ensure accommodation, the mice were allowed to swim freely for 2 min without the platform in the pool on the day before the experiment. The MWM experiment is composed of two tests: (1) spatial learning test (lasting 5 days) in which each mouse was put into the pool every day and the time required for the mouse to find the platform was recorded as the latency (in seconds; if the mouse could not find the platform within 120 s, it was placed on the platform for 30 s) and (2) probe test (on day 6) in which each mouse was placed at a specific location in the pool (with the platform removed) and the movement of the mouse was recorded and analyzed.

### 2.8. RT-PCR

The total RNA was isolated from the brain using Trizol, and the ratio of A260/A280 values was employed to quantify the concentration of RNA. cDNA synthesis and quantitative PCR were performed following a previous report [52]. The GAPDH was employed as a housekeeper gene, and the relative expression levels of MAPK1 and GAPDH genes were calculated by the 2^−△△ct^ method. Target primer sequences of MAPK1 were provided by Vazyme (Vazyme, Nanjing, China) as follows (5′–3′): MAPK1 (GCACCAACCATCGAGCAAAT and CTTGAGGTCACGGTGCAGAA).

### 2.9. Immunofluorescence

Immunofluorescence experiments were employed to investigate the protein level of phosphorylated mitogen-activated protein kinase 1 (pMAPK1) in the hippocampus and spinal dorsal horn of the mice following a previously described method [53]. After the behavioral tests, the mice were anaesthetized with an intraperitoneal injection of 1% sodium pentobarbital (50 mg/kg) and then perfused with saline and 4% paraformaldehyde. After that, the brain and spine were carefully harvested and frozen in liquid nitrogen. Hippocampus and spinal dorsal horn samples were cut into 4-*μ*m sections, permeabilized with 0.2% Triton X-100 in phosphate-buffered saline, and blocked using a blocking buffer. A primary antibody against pMAPK1 (p-ERK) (ab201015, 1 : 200; Abcam, Cambridge, UK) was added and the sections were incubated overnight at 4°C followed by incubation with an Alexa Fluor® 555-conjugated secondary antibody. Images were then captured using an immunofluorescent camera (3DHISTECH Ltd., Budapest, Hungary). The cornu ammonis 1 and 3 (CA1 and CA3) regions in the hippocampus were determined following a previously described method [54]. The relative fluorescence density of the target protein within a fixed area was determined using Image-Pro Plus v6.0.

### 2.10. Statistical Analysis

The network pharmacology and molecular docking data were generated by software or databases as mentioned above. The experimental data were expressed as mean ± SD if not otherwise stated. The statistical analysis was performed in SPSS v27 first with an orthogonality test and homogeneity test of variance, followed by the one-way analysis of variance (ANOVA) and the least significant difference (LSD) or Tukey's post hoc test (*n* = 6). *P* < 0.05 was considered significant. The results were visualized using GraphPad Prism v6.

## 3. Results

### 3.1. Prediction of Target Proteins

**Figure 2:**
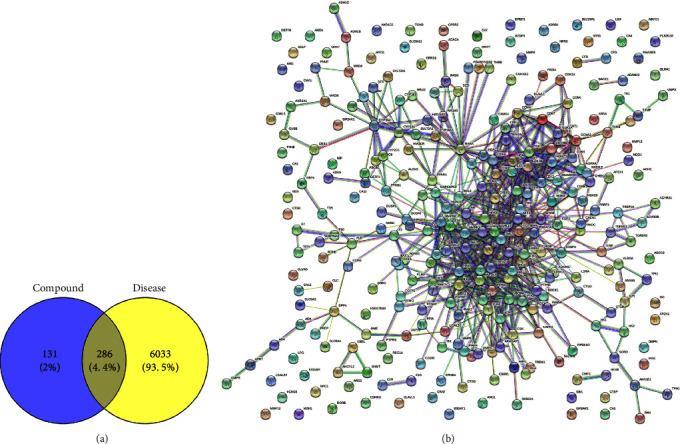
The network pharmacology analysis results. (a) Venn diagram of compound orientin and related genes. (b) The PPI network based on overlapped target genes.

After removing the duplicates, 417 predicted targets of orientin were obtained from the abovementioned databases. In addition, 6322 target proteins involved in neonatal pain were obtained from the abovementioned databases. The 286 overlapping targets are shown in a Venn diagram in Figure 2(a).

### 3.2. PPI Network

The STRING database was used to construct a PPI network of the 286 proteins. As shown in Figure 2(b), there were 286 nodes and 762 edges, with a mean node degree of 5.08. The proteins were grouped into one cluster, with a few independent, unrelated proteins. There were many interactions among the proteins in the cluster, and the principal proteins were CDK4, MAPK14, and MAPK1. The cytoHubba plugin in Cytoscape was used to determine the following top 15 hub proteins (ranked by degree) in the PPI network: SRC (with a score of 41), followed by HSP90AA1 (37), MAPK1 (36), RHOA (28), EGFR (27), AKT1 (27), PTPN11 (27), ESR1 (26), RXRA (26), HRAS (26), MAPK14 (24), MAPK8 (22), HDAC1 (22), VEGFA (21), and JAK2 (21).

### 3.3. GO and KEGG Enrichment Analyses

**Figure 3:**
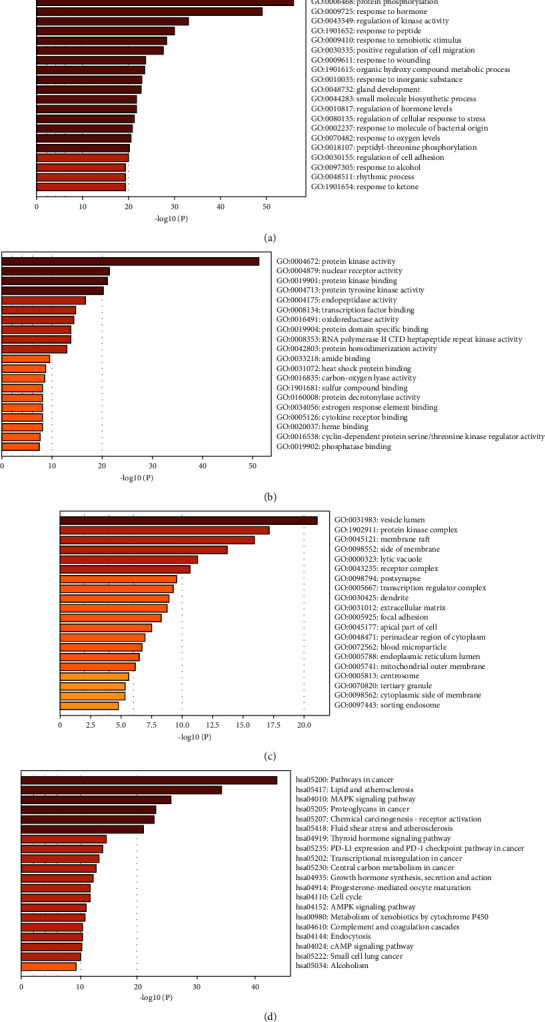
The GO and KEGG prediction results. (a) The BP results of GO enrichment analysis. (b) The MF results of GO enrichment analysis. (c) The CC results of GO enrichment analysis. (d) The KEGG prediction results.

In the GO analysis (minimum overlap = 3, *P* < 0.01, and minimum enrichment = 1.5), there were 260 BPs, 120 MFs, and 98 CCs. The top 20 BPs, MFs, and CCs (ranked by −log_10_ (*P* value)) are shown in Figures 3(a)–3(c), respectively. The highest-ranked BP, MF, and CC were protein phosphorylation, protein kinase activity, and vesicle lumen, respectively.

In the KEGG analysis, 157 pathways were predicted. The top 20 pathways (ranked by −log_10_ (*P* value)) are shown in Figure 3(d). Among the top 20, several apparently unrelated pathways were disregarded (e.g., pathways in cancer), and the mitogen-activated protein kinase (MAPK) signaling pathway mostly attracted our interest.

### 3.4. Molecular Docking Analysis

**Figure 4:**
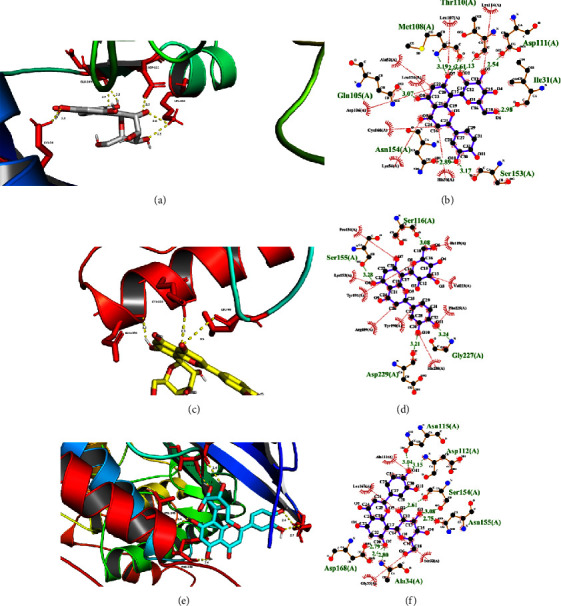
The molecular docking results. (a) The locally enlarged picture of orientin bonding to 7E73 (MAPK1). (b) The two-dimensional picture of orientin bonding to 7E73 (MAPK1). (c) The locally enlarged picture of orientin bonding to 3VUM (MAPK8). (d) The two-dimensional picture of orientin bonding to 3VUM (MAPK8). (e) The locally enlarged picture of orientin bonding to 3FLZ (MAPK14). (f) The two-dimensional picture of orientin bonding to 3FLZ (MAPK14). Amino acids are shown in red and hydrogen bond distances are shown in yellow dashed lines (unit: Å).

As the MAPK signaling pathway may be key, the docking of orientin with the following three MAPK-related proteins was investigated: MAPK1 (PDB ID: 7E73), MAPK8 (PDB ID: 3VUM), and MAPK14 (PDB ID: 3FLZ). As shown in Figures 4(a)–4(c), orientin exhibited tight binding with all three target proteins, with >1 hydrogen bond, suggesting that orientin can strongly associate with these proteins. The binding energies were low, at −8.1, −5.1, and −8.2 for MAPK1, MAPK8, and MAPK14, indicating stable compound-protein binding (threshold: <−5 kcal/mol) [55].

### 3.5. Effect of Orientin on Mechanical and Thermal Pain Thresholds in Adult Mice

**Figure 5:**
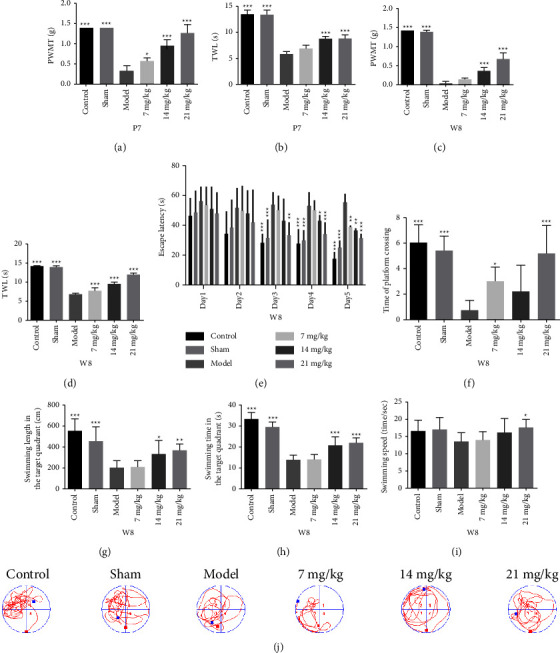
The experiment validation of pain. (a) The PWMT of mice at day 7 (P7). (b) The TWL at day 7 (P7). (c) The PWMT at week 8 (W8). (d) The TWL at week 8 (W8). The experiment validation of cognition at week 8 (W8). (e) The escape latency in the spatial learning phase (s). (f) Numbers of crossings of the platform. (g) The swimming length in the target quadrant (cm). (h) The time spent in the target quadrant (s). (i) The swimming speed (s). (j) The swimming track diagram during the probe test on day 6 (the probe test phase). The red dot is the starting point, the blue dot is the ending point, and the original platform position is a red circle in quadrant 2. Significant differences between model groups and other groups are indicated as ^*∗*^*P* < 0.05, ^*∗∗*^*P* < 0.01, and ^*∗∗∗*^*P* < 0.001.

As shown in Figures 5(a) and 5(b), at P7, PWMT and TWL were significantly decreased in the model group compared to the control group, indicating immediate hyperalgesia in neonatal mice. In addition, PMWT was increased in two orientin groups (14 and 21 mg/kg) and TWL in all three orientin groups compared to the model group. PMWT and TWL were not different in the sham group compared to the control group, indicating that gentle touch did not elicit neonatal pain.

As shown in Figures 5(c) and 5(d), at W8 (after reinjury), PWMT and TWL were significantly decreased in the model group compared to the control group, indicating long-term hyperalgesia in adult mice that experienced repeated procedural neonatal pain. PMWT was increased in all three orientin groups and TWL in two orientin groups (14 and 21 mg/kg) compared to the model group.

### 3.6. Effect of Orientin on Cognition in Adulthood

As shown in Figure 5(e), in the spatial learning test, the escape latency was not significantly different in the model group compared to the other groups on day 1. However, on days 3–5, the escape latencies were significantly increased in the model group compared to the control group and significantly reduced in the orientin groups compared to the model group (21 mg/kg groups on days 3–5, 14 mg/kg groups on days 4–5, and 7 mg/kg group on day 5). During the probe test on day 6, platform crossings decreased and the time spent on the target quadrant decreased in the model group compared to the control group. However, in the orientin groups compared to the model group, the number of platform crossings increased (7 and 21 mg/kg groups) (Figure 5(f)) and the swimming length and time spent on the target quadrant increased (14 and 21 mg/kg groups) (Figures 5(g) and 5(h)). There were no significant differences between the control and sham groups. Swimming speeds are shown in Figure 5(i) while representative traces are shown in Figure 5(j).

### 3.7. *In Vivo* Validation of Mechanism of Action

**Figure 6:**
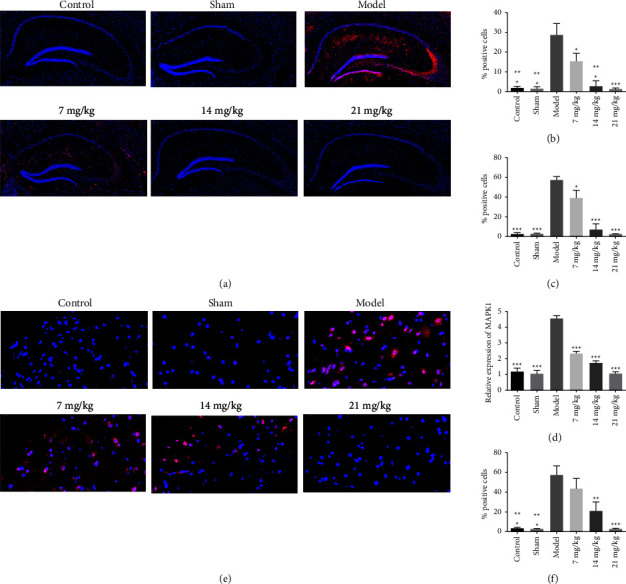
The intrinsic mechanical experiment validation. (a) The pMAPK1 (p-ERK) levels in the hippocampus of the brain and quantification in the CA1 region (b) and quantification in the CA3 region (c). Inner scale bar at top left corner = 100 *μ*m. (d) The MAPK1 gene expression in the brain. The pMAPK1 levels in the spinal dorsal horn (e) and quantification (f). Inner scale bar at top left corner = 20 *μ*m. Significant difference between model groups and other groups were indicated as ^*∗*^*P* < 0.05, ^*∗∗*^*P* < 0.01, and ^*∗∗∗*^*P* < 0.001.

As shown in Figure 6(a), the pMAPK1 (p-ERK) protein levels in the hippocampus (quantification of CA1 region in Figure 6(b) and CA3 region in Figure 6(c)) were increased in the model group compared to the control group and decreased in the orientin group compared to the model group. The gene expression of MAPK1 showed a similar tendency in Figure 6(d). The pMAPK1 protein levels in the spinal dorsal horn exhibited similar patterns, as shown in Figures 6(e) and 6(f).

## 4. Discussion

Although researchers have found that orientin has multiple pharmacologic effects, this is the first study to show that orientin inhibited the long-term hyperalgesia and cognitive impairment elicited by repeated procedural neonatal pain. Network pharmacology and molecular docking analysis predicted that the MAPK signaling pathway may be involved. In particular, the pMAPK1 levels in the central nervous system of mice play crucial roles in orientin's effect.

The behavioral changes in adult mice that were exposed to repeated procedural pain as neonates have attracted many pediatric researchers' interest. Neonatal pain elicits a range of immediate and long-term adverse effects in neonates and adults. Clinical observations revealed the mechanical hyperalgesia and conditioning of the pain response [56]. An increase in inflammatory cytokines such as interleukin-6 (IL-6), interleukin-1*β* (IL-1*β*), and tumor necrosis factor-*α* (TNF-*α*) was found in rats with repeated procedural neonatal pain; these cytokines participate in central sensitization and hyperalgesia development and maintenance [57]. MAPK is tightly linked with neuroinflammation and cytokine production [58] and is involved in a positive feedback mechanism involving excessive spinal dynorphin expression after peripheral noxious stimulation [59, 60]. Inhibition of MAPK in a neuropathic pain model downregulated the excessive production of cytokines in the spinal dorsal horn [61]. In our study, the phosphorylation of MAPK1 in the dorsal horn likely played crucial roles in orientin's effect on neonatal pain immediately and in the long-term in adults.

Another important adverse effect of repeated procedural neonatal pain is cognitive impairment. Inflammatory pain in the early life of rats elicits long-term deficits in the hippocampal-dependent spatial memory [62]. A well-acknowledged mechanism of neonatal pain-induced adult changes is the activation of the hypothalamic-pituitary-adrenal (HPA) axis and related changes in the hippocampus [63, 64]. In premature infants in neonatal intensive care units, the frequency of skin incision/puncturing procedures is closely related to high cortisol in later life [65]. Another report on rats revealed that neonatal pain elicited immune activation involving both spinal cord neurons and the HPA axis [66]. The HPA axis is linked to the MAPK signaling pathway, and this pathway is a key pathway regulated in the hippocampus in both acute and chronic stress [67]. Intracerebroventricular injection of corticotropin-releasing hormone in mice increased pMAPK1 in hippocampal CA1-CA3 areas [68], while it reduced pMAPK (P38 and ERK) levels in rats which improved cognitive impairment [69]. In the present study, inhibition of MAPK1 in the hippocampus likely played a role in orientin's preventative effect regarding cognitive impairment.

This study has several strengths and limitations. The primary strength is that we reported a new application, i.e., pediatric pain management, of a well-known and well-tested agent. Animal research on orientin has been conducted for over 2 decades [70]. Multiple studies indicate that orientin is a relatively safe compound [23, 71]. Our study suggested another natural product for neonatal pain, although there is a huge gap from preclinical to clinical. Another major finding is that the MAPK signaling pathway is not only activated in the spinal dorsal horn after repeated procedural pain in neonatal mice but it is also activated in the hippocampus, which has not been reported in previous studies. The phosphorylation of MAPK1 in the hippocampus has been suggested to be responsible for depression, cognitive decline, and other symptoms in adulthood in animal models of repeated procedural neonatal pain [59]. In addition, we reported the link between orientin and MAPK1 in a new organ, i.e., the brain. Previous experiments involving extracts containing orientin revealed that orientin regulated the MAPK signaling pathway [72, 73], and other studies suggested that SRC and MAPK play a crucial role in orientin's antiapoptosis ability [74] and p38 MAPK was involved in orientin's antioxidative stress effect [75]. However, most of these results came from *in vitro* models [72–74]. The current study revealed that orientin regulated MAPK1 activity in the brain. Supplementation with orientin-enriched food, such as fruit, may be an alternative strategy for pain management in premature neonates.

In contrast, the primary study limitation is the lack of an agonist/inhibitor of MAPK signaling used in animal experiments. Orientin is a highly water-soluble flavone and can exert effects on the brain [76, 77]. In contrast, most agonists for use in the brain require intracerebroventricular injection, and although we tried several approaches for intracerebroventricular injection in newborn pups in preliminary experiments, all attempts failed. Therefore, an agonist/inhibitor of MAPK signaling pathway was not used in animal experiments so there was a lack of validation of the role of MAPKs.

Some aspects which should be paid more attention to in future studies are as follows. First, there are many types of chronic pain in pediatric patients, for example, postoperative pain, abdominal pain, repetitive operational pain, nociceptive pain, and low back pain [78, 79]. However, the mechanisms of chronic pain are commonly similar, mainly caused by the complex interaction between primary afferent nerves, dorsal horn neurons, spinal glia, and brain [78]. Central sensitization, several signaling pathways, and neuroinflammatory genes contributed to the pain [80]. Among them, nuclear factor kappa B (NF-*κ*Β), MAPK, and inflammasome NOD-like receptor (NLR family) pyrin domain containing 3 NLRP3 play important roles in neuroinflammation and pain [81]. Flavonoids are natural compounds, found in fruits, vegetables, and various dietary sources. Flavonoids have been widely used for their analgesic effect due to their anti-inflammatory and antioxidant abilities. For example, quercetin modulated the MAPK, NF-*κ*B, and NLRP3 to alleviate the inflammatory pain, neuropathic pain, and cancer pain [82]. Orientin can modulate the MAPK and NF-*κ*B [24], therefore it can be used for other chronic pain treatments in pediatric patients. Second, there is a huge gap between preclinical and clinical experiments. Current studies on orientin were animal studies [83, 84], therefore the pain alleviation effect and side effect on humans are unknown. Fortunately, the structure of orientin is similar to luteolin [85], while luteolin has been tested in many clinical trials. Luteolin is effective in pain inhibition [81], with above 5,000 mg/kg LD50 values in rats [86], and safe in pediatric patients [87]. The orientin is C-glycoside while luteolin is aglycone [88]. Compared with aglycone, a glycoside is commonly a more suitable drug because it can improve stability, increase water solubility, reduce toxicity, and most importantly, enhance the specific targeting properties of drugs [88]. Therefore, it can be predicted that orientin is an effective and relatively safe drug. Moreover, to mimic the gap, strict clinical trials on dose and the side effects are critical in the future. Third, orientin research studies may develop in the following directions. For preclinical research, current research studies on pain mediators and regulators were gradually enriched, and research studies on new pathways and mechanisms have increased. However, research on the mechanism of orientin in neonatal pain was limited. Therefore, it is necessary to expand the investigation on new mechanisms of orientin in neonatal pain. For clinical research, research on its effectiveness, safety, and drug metabolism, especially metabolism, in the central nervous system of newborns and infants will be critical.

## 5. Conclusion

Based on network pharmacology, molecular docking analysis, and experimental validation, this study revealed that orientin alleviated repeated procedural neonatal pain and improved the long-term cognitive deficit. The MAPK signaling pathway plays a crucial role in the effect of orientin. Molecular docking analysis predicted that orientin can bind tightly to the target MAPK proteins. This study provided new insights into the potential application of a natural flavonoid for neonatal pain treatment.

## Data Availability

The data used to support the findings of this study are available from the corresponding author upon request.
